# Novel Ni/Zn MOFs for Sorbitol Production via Catalytic Transfer Hydrogenation

**DOI:** 10.3390/molecules30234565

**Published:** 2025-11-27

**Authors:** Vuyolwethu Tokoyi, Nirmala Deenadayalu

**Affiliations:** Department of Chemistry, Faculty of Applied Sciences, Durban University of Technology, Durban P.O. Box 1334, South Africa; nirmalad@dut.ac.za

**Keywords:** catalytic transfer hydrogenation (CTH), metal–organic framework (MOF), glucose, sorbitol, and sacrificing alcohols

## Abstract

Researchers continue to explore alternative catalysts that are more abundant and effective for hydrogenation reactions to produce sorbitol, such as those using nickel, ruthenium, or zirconium metal centers. This study examined the catalytic transfer hydrogenation of glucose to sorbitol using different alcohol hydrogen donors, specifically ethanol, isopropanol, 1,4-butanediol, and 1,4-cyclohexanediol, with the prepared Ni/Zn MOF catalysts. It also assessed how sacrificial alcohols affected the transformation and selectivity toward sorbitol. The results confirmed the successful catalytic activity and feasibility of this process using MOFs, especially the Ni-based one, which produced up to 51.8% sorbitol, while the Zn-based catalyst yielded 42.3% sorbitol in 1,4-cyclohexanediol. Sacrificial diols exhibited enhanced efficacy as hydrogen donors relative to short-chain alcohols, specifically terminal diols such as 1,4-butanediol and 1,4-cyclohexanediol, which provided substantial hydrogen donation potential and improved selectivity in the conversion of glucose to sorbitol, achieving maximum yields of 45.12% with 1,4-butanediol and 51.8% with 1,4-cyclohexanediol. Regarding the catalysts, both Ni and Zn MOFs improved the transfer hydrogenation process in sugar alcohol mixtures compared to aqueous solutions, and particularly the Ni MOF, with its high surface area and multiple active sites, enhanced the catalytic transformation process. The results clearly indicate that the structural and chemical properties of these alcohols affect the quantity of hydrogen generated and transferred, which is crucial for the efficient overall yield of sorbitol. This insight enhances the understanding of this engineered system and its potential future applications in sustainable biomass utilization.

## 1. Introduction

Sorbitol, a valuable sugar alcohol with applications in food, pharmaceuticals, and chemical synthesis, is conventionally produced through the catalytic hydrogenation of glucose using hydrogen gas over metal catalysts, such as nickel or ruthenium [1]. However, this process often requires high hydrogen pressure, it is relatively complicated, has a low level of safety, and can suffer from catalyst deactivation and low selectivity, which may lead to less efficiency [2]. However, the use of hydrogen gas is not the only way to do hydrogenation; the process of catalytic transfer hydrogenation (CTH) offers a safer and potentially more sustainable alternative, utilizing liquid hydrogen donors such as alcohols [3] or diols instead of gaseous hydrogen [4].

The hydrogenation of glucose to sorbitol often employs transition metal-based catalysts, since these metals exhibit both Brønsted and Lewis acidity, including active sites that facilitate high glucose conversion rates and favorable selectivity for sorbitol [5,6]. In the synthesis of biochemicals, numerous researchers employ abundant inorganic compounds as catalysts in the hydrogenation process. These include metal alloys such as iron-nickel (Fe-Ni) [7], Ni/bentonite [8], nickel-aluminum (Ni-Al) [9], Ni/SiO_2_ [10], and Raney Nickel [11], which have been utilized in the hydrogenation of glucose to sorbitol under elevated hydrogen pressure. While various catalysts have been investigated for this transformation, their practical application is often hampered by limitations such as low surface area, poor recyclability, and the requirement for high hydrogen pressures [12,13]. In contrast, metal–organic frameworks (MOFs) offer a unique platform to address these challenges by providing high surface areas for better dispersion of active sites, tunable pore environments that can enhance catalytic efficiency of the materials, providing ample active sites, and facilitating mass transfer [1,6,14,15]. These attributes position MOFs as compelling candidates for designing bifunctional catalysts that combine acid and redox functionalities for efficient glucose conversion into sorbitol [16]. For instance, the intrinsic Lewis acidity of certain MOFs, such as those incorporating zirconium (Zr), can facilitate the initial dehydration of glucose, while strategically introduced functional groups like amino or sulfonyl moieties can further assist in isomerization or act as Brønsted acid sites, respectively [17]. The superior results observed and reported regarding some MOF applications, particularly those utilizing large-pore structures like MIL-101 in the isomerization of glucose to fructose, further underscore their potential in catalytic performance [18,19].

The application of MOFs has gained popularity in catalysis as catalysts or catalyst supports that can be used in several reactions, and this is attributed to the control of the MOF architecture, which allows for the rational design of these materials with optimized pore sizes and functionalities, facilitating the selectivity potential towards biochemicals [17]. As a result of these properties, these materials have been utilized as hosts for acidic or nanomaterial catalysts in the hydrogenation process of glucose to sorbitol production.

Chen et al. [6] utilized a MOF as a host for phosphotungstic acid (PTA) and a ruthenium nanoparticle (Ru-NP) multifunctional catalyst to produce a multifunctional catalyst that combined the PTA-catalyzed hydrolysis of cellulose to glucose with subsequent ruthenium metal-catalyzed hydrogenation, resulting in sorbitol as the desired product. A MOF was chosen as support because the phosphotungstic acid (PTA = H_3_PW_12_O_40_) loading could be controlled effectively by using the “ship in a bottle” approach. A sorbitol yield of 57.9% was achieved by adjusting the ratio of acid (PTA) and ruthenium nanoparticle metal catalyst. Importantly, the MOF alone showed no significant catalytic activity. Only very low amounts of glucose (0.5%), ethylene glycol, and glycerol (4.1% combined) were measured. Herbst and Janiak [20] achieved a remarkable yield of 95.1% sorbitol using cellobiose as a feedstock with the Ru/PTA@MIL-100Cr catalyst. In contrast, reference experiments employing only MIL-100Cr or PTA@MIL-100Cr yielded lower quantities of glucose and ethylene glycol/glycerol (13.5%, 7.2% and 11.3%, 5.6%, respectively). The Ru@MIL-100Cr catalyst produced a sorbitol yield of 56%, underscoring the necessity for a meticulous balance of acidity.

In a CTH process for sorbitol production, the catalyst is generally suspended in a solution comprising glucose and the hydrogen donor under heating conditions [3,4]. The transformation process involves the catalyst activating the hydrogen donor, generating active hydrogen species on the catalyst surface. Due to MOFs’ well-known high catalytic activity, these adsorbed hydrogen atoms subsequently react with the adsorbed glucose molecules, reducing them to sorbitol. They may also have an impact on the reaction pathway through their Lewis or Brønsted acidic sites or the basic site from the organic linkers and metal centers [21]. Furthermore, while research in this area is still ongoing, studies have demonstrated the potential of Ni-based catalysts in the hydrogenation process for the efficient and selective production of sorbitol from glucose under milder reaction conditions compared to using traditional catalysts. In this study, Ni and zinc (Zn) ZIF-8 MOFs were prepared and evaluated as catalysts in the CTH process for converting glucose to sorbitol in an alcohol medium.

## 2. Results and Discussion

### 2.1. Spectroscopic Data

The synthesized materials were characterized using powder XRD, FT-IR, UV-Visible DRS, and SEM methods, and the observed results are in line with the reported results (Figures S1–S3). The recorded electronic spectra of MOFs in toluene exhibit bands assigned to charge transfer transitions (CCT) and d-d transitions. The transitions within the d-orbitals of the Ni^2+^ ion observed at 352–428 nm and 396–698 nm are attributed to the Ni-S and Ni-N bonds. In the visible region of the spectra for Ni MOF, absorption bands presented in Figure S1c are attributed to [^3^A_2_g → ^3^T_1_g(P) (ν_3_)] and [^3^A_2_g → ^3^T_1_g(F) (ν_2_)] d-d transitions [22,23], while, for Zn MOF, the Zn^2+^ *d*-orbitals are filled [24] and the observed absorption band is attributed to metal to charge transfer transition (MLCCT) [25].

[M^1^: Zn:2-MBT]: Pale yellow solid; Yield: 72%; DLS: d_h_ = 1066.0 nm; FTIR (**ν_max_/**cm^−1^): 3051 (arC–H), 1509 (C-N), 1431 (N=C), 731 (-C-S); UV-Vis (Toluene): (λ_max/_ nm): 280–316 nm [Metal-to-Ligand Charge Transfer Transition (MLCTT)].

[M^2^:Ni:2-MBT]: Brown solid; Yield: 69%; DLS: d_h_ = 379.4 nm; FTIR (**ν_max_/**cm^−1^): 3123 (arC–H), 1430 (C-N), 1231 (N=C), 681 (-C-S); UV-Vis (Toluene): (λ_max_/nm): *d*-*d* transitions **→** 352–428 nm [^3^A_2_g → ^3^T_1_g(P) (ν_3_)], 396–698 nm [^3^A_2_g → ^3^T_1_g(F) (ν_2_)].

### 2.2. SEM Images and EDX Elemental Mapping

**Figure 1:**
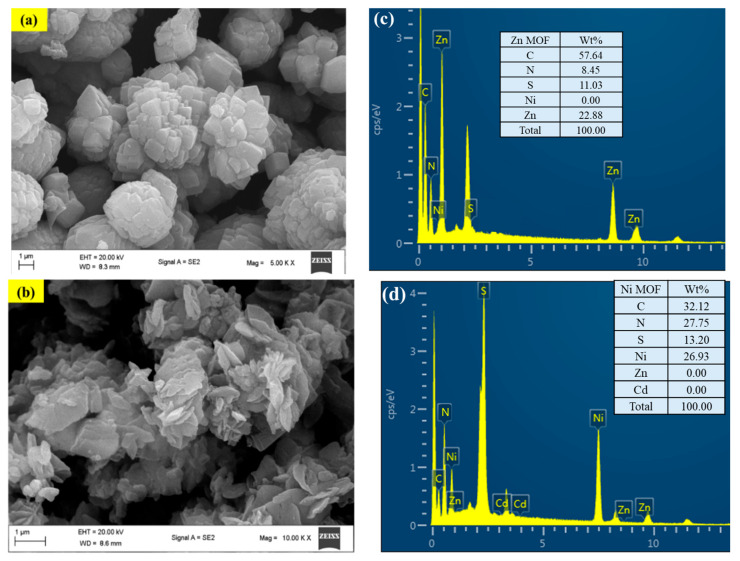
SEM images of (**a**) Zn MOF, (**b**) Ni MOF, and (**c**) and (**d**) EDS profiling of Ni/Zn MOFs.

The surface morphologies, microstructure, and particle distribution of the synthesized Zn/Ni MOFs were characterized using field emission scanning electron microscopy (FESEM). The Zn MOF exhibited a constant particle size, and its distribution was relatively uniform (Figure 1a). Upon further magnification, the image revealed conglomerated, distorted plate-like structures in the form of microspheres. Figure 1b illustrates that Ni:2-MBT MOF has a rough surface with particles observed in the 1 μm image appearing as a plate-like structure, which were found to be widely and non-uniformly distributed on the surface. Furthermore, to gain more insights into the purity, chemical composition, and spatial distribution of elements on the surface of the MOFs, elemental mapping was employed using energy-dispersive X-ray spectroscopy (EDX) analysis. Figure 1c shows an EDX profile of carbon (C), nitrogen (N), sulphur (S), zinc (Zn), and nickel (Ni) elements to be present within the compounds, confirming that there were no impurities in the produced MOFs, which can be attributed to the homogeneous arrangement of components within the materials.

### 2.3. Particle Size Distribution and BET Analysis

Dynamic light scattering (DLS) and BET analysis were performed to determine the hydrodynamic size and size distribution of the synthesized MOFs, specific surface area, pore volume, pore width, and pore-size distribution of the MOF catalysts. The analysis revealed a hydrodynamic diameter (d_h_) of 1066.0 nm for Ni MOF and 379.4 nm for Zn MOF. The intensity-weighted size distribution graphs depicted in Figure S3 showed a single, sharp peak for each MOF within the nanometer range, further corroborating the monodispersity of the particles within the materials, and this distribution is critical for ensuring predictable behavior in several areas, like catalysis.

Figure S4a demonstrated that the adsorption isotherms of all samples were type II (with an H3 hysteresis loop, suggesting the existence of microporous-mesoporous nature in samples, according to IUPAC classification [26]. Utilizing the BET straight line fit (Figure S4b), the obtained surface area for Ni MOF was higher (136.59 m^2^/g) than that of Zn MOF (113.84 m^2^/g), with a pore volume of 0.62 cm^3^/g for Ni MOF and 0.35 cm^3^/g for Zn MOF, indicating a strong physisorption property.

### 2.4. Bronsted-Lewis Acid Sites Estimation

Pyridine and 2,6-lutidine adsorption FTIR spectroscopy were performed to gain a deeper insight into the Lewis and Brønsted acid sites of the synthesized MOFs [27,28]. After pyridine adsorption (Figure S5a), the spectra of the MOFs exhibit characteristic bands at 1446 and 1487 cm^−1^ for Ni MOF, 1454 and 1489 cm^−1^ for Zn MOF, indicating the interaction between pyridine and metal sites, specifically, the Lewis acid sites [29]. Figure S5b illustrates two split bands, 1029.82 and 1018.25 cm^−1^, for Zn MOF, and three bands, 1031.75, 1012.46, and 987.39 cm^−1^, for Ni MOF, which correspond to the interactions formed between 2,6-lutidine and the Brønsted acid sites from the thiol (-SH) and thione (N-H) forms in the organic ligands.

### 2.5. Thermal Stability

**Figure 2:**
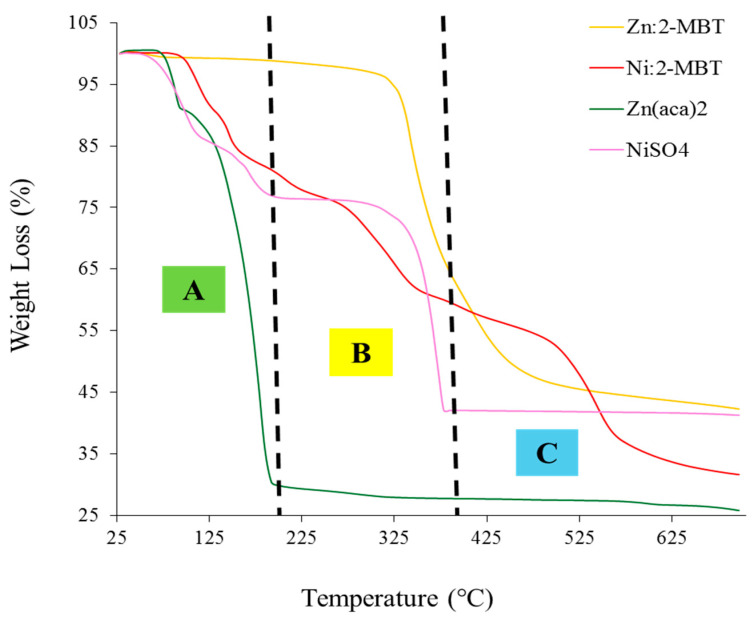
Superimposed TGA thermograms of Ni and Zn MOFs.

Thermogravimetric analysis (TGA) shows that the Zn/Ni MOFs have three weight loss regions. Figure 2 shows the TGA profiles of the heat-treated catalysts in the temperature range from 25 °C to 800 °C. The first two weight losses (~10% and ~5%) observed in section A for the Ni MOF sample are attributed to the endothermic reaction process that removed methanol and water solvents contained within the material, which were used for washing and synthesis. In Section B, the approximately 50% weight loss at 322 °C is attributed to the volatilization of the surplus organic linker 2-mercaptobenzothiazole (2-MBT) in the Ni MOF [30]. The steep and sharp drop observed for the Zn MOF between 218 °C and 403 °C, with a mass loss of about 65%, can also be attributed to 2-MBT volatilization, since its melting point is within 177–181 °C. Both steps of thermal degradation are exothermic reactions, and the thermal degradation ends at 525 °C. Beyond 525 °C, residual Ni MOF (~28%) and Zn MOF (~47%) were recorded due to framework degradation [31], leading to the formation of NiO and ZnO [32], and potential destruction of M-S or M-N bonds. According to these results, both MOFs are thermally stable and in line with the literature [33]. The TGA profiles also illustrate that Zn MOF is more stable than Ni MOF, which can be attributed to the formed M-S bonds, which often contribute to the insolubility and chemical robustness, further resisting hydrolysis and the chemical decomposition of the overall framework [34,35].

### 2.6. PXRD Patterns

The XRD patterns of the synthesized MOFs, shown in Figure S6a,b, demonstrated diffraction peaks almost the same as the simulated peaks but with a higher intesity. This similarity suggested that these materials maintain a high level of crystallinity despite the introduction of different metal salts. The main diffraction peaks observed in the prepared materials were located at 2Ө = 6.77°, 9.79°, 11.74°, 17.45°, 19.54°, and 27.93° for Ni MOF, 7.33°, 10.42°, 12.75°, 14.62°, 16.47°, and 18.56° for Zn MOF, respectively. Interestingly, the peak intensities for the synthesized Ni MOF compared to those of Zn MOF were higher, and this indicated that Ni MOF had a higher degree of crystallinity than Zn MOF. These findings agree well with the successful synthesis of the MOFs. Notably, no additional peaks corresponding to impurities or secondary phases were detected, confirming the high phase purity of the synthesized catalysts.

### 2.7. XPS

**Figure 3:**
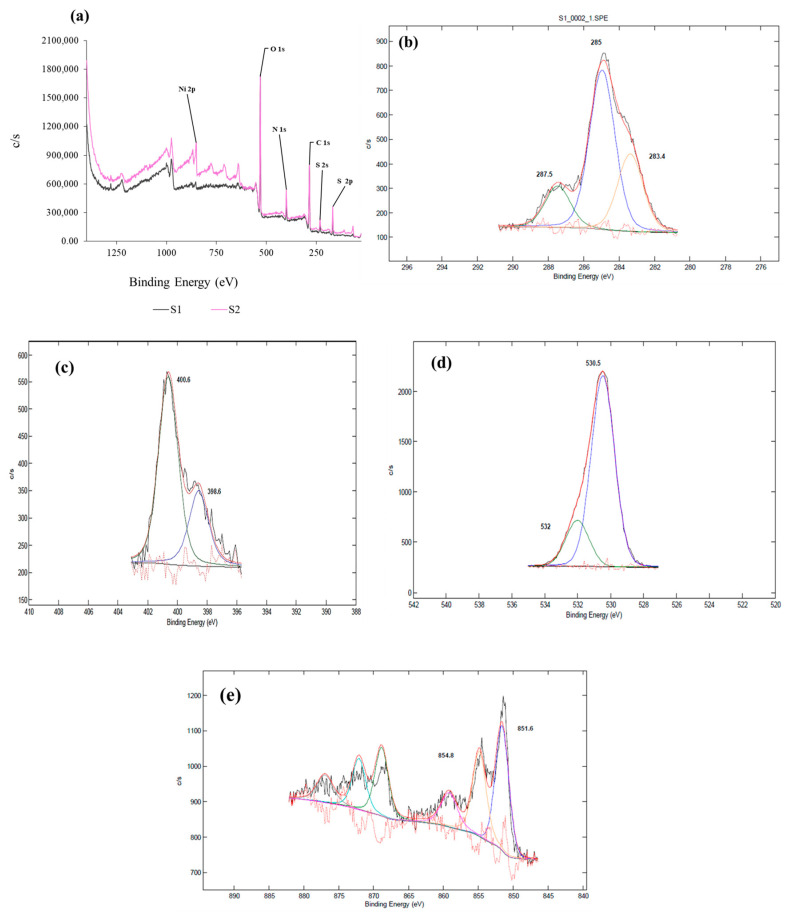
XPS spectra of Ni-MOF: (**a**) survey spectra, (**b**) C1s, (**c**) N1s, (**d**) O1s, and (**e**) Ni 2p (red line: total fitted curves-sum of all component peaks, black line: raw experimental, blue and green lines: component peaks for C, O, and N).

The Ni MOF catalyst, which was shown to be more efficient than Zn MOF, was further studied by XPS to determine the chemical state of the surface and the Ni MOF’s elements. Figure 3a illustrates an XPS survey spectrum of the prepared Ni MOF catalyst with registered C, O, N, S, and Ni components on the surface of the catalyst. The spectra of C1s (Figure 3b) show three different peaks at 283.4, 285, and 287.5 eV, and these are attributed to the bonds of C-C, C-N/C=N, and C-S, respectively [36]. Hybrids of cobalt/iron phosphides derived from bimetal–organic frameworks as highly efficient electrocatalysts for oxygen evolution reaction. Figure 3c shows two peaks at 400.6 and 398.6 eV attributed to the 2-MBT ligand that contributes to the two distinct chemical environments for nitrogen, with the difference in binding energy primarily caused by coordination versus non-coordination to the Ni ion [37,38].

The spectra of O1s (Figure 3d) have two peaks at 530.5 and 532 eV, peaks typically attributed to the presence of surface-adsorbed or hydrated species, such as H_2_O and OH^−^, which are common contaminants [39,40,41]. The presence of bound hydroxyl groups enhances water binding [42], as the H_2_O–OH H-bonding is stronger than the H_2_O–H_2_O H-bonding [43], resulting in the obtained binding energy due to the more covalent nature of the oxygen bonds in water and hydroxide groups, where the oxygen atom experiences a lower electron density compared to the lattice oxygen [44]. These can also be caused by anionic impurities (SO_4_^2−^) or oxidized sulfur species with low binding energy. The Ni 2p spectra (Figure 3d) show peaks at 851.6 and 854.8 eV, indicating spin energy owing to Ni 2p_3/2_ and Ni 2p_1/2_ spin-orbits, which correspond to the MOF structure and Ni-OH, respectively [41,45,46]. The binding energy peaks also recorded from around 858.3 (2p_3/2_) to 878.4 eV were ascribed to the satellite peaks of Ni 2p [36,46]. The outcomes above show the characteristics of Ni^2+^ and prove the successful formation process of the nickel 2-mercaptobenzothiazole phases.

### 2.8. Catalytic Results

#### 2.8.1. Produced Biochemicals

MOFs are often used as catalyst precursors or supports for active metal nanoparticles, which are then applied to hydrogenation reactions of glucose to sorbitol, but this study evaluated the capability of Ni and Zn MOF catalysts to catalytically transform glucose into sorbitol in the presence of different alcohols, namely, ethanol, isopropanol, 1,4-cyclohexanediol, and 1,4-butanediol as solvents, with the 1,4-butanediol solvent previously reported to have an impact on the CTH of glucose to sorbitol through hydrogen donation [3,4]. For these reasons, the above-mentioned alcohols were used as potential hydrogen donors in the CTH process in the presence of MOF catalysts. Table 1 below presents information on catalysts previously used in relation to hydrogenation or CTH processes that are relevant to the glucose-to-sorbitol transformation, along with a key study reference point of using Raney Nickel in CTH as a comparison.

**Figure 4:**
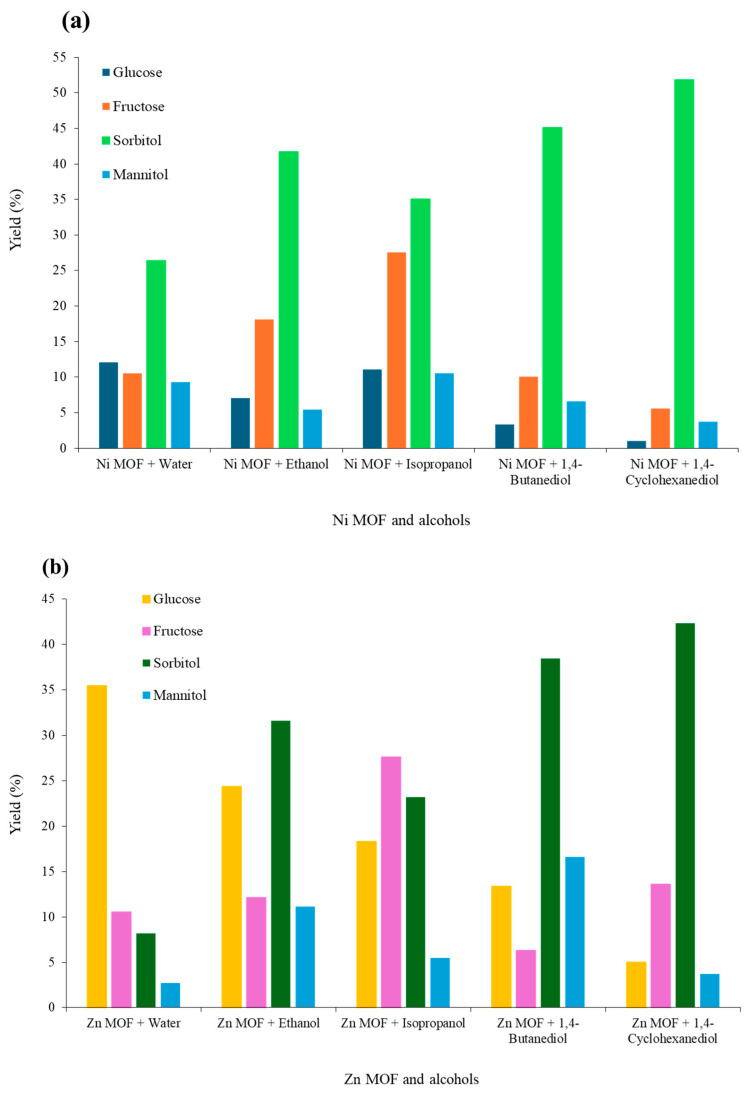
Product yields obtained from the CTH of glucose in different solvents in the presence of (**a**) Ni MOF and (**b**) Zn MOF catalysts. Reaction conditions: temperature = 150 °C; glucose = 2 g; reaction time = 6 h.

The results shown in Figure 4, compared to the yield from glucose transformation using water or with catalysts only, or with a catalyst + alcohol, demonstrated that glucose conversion was successful, with high yields produced under both with- and without-solvent conditions. Fructose (10.56%) and sorbitol (26.40%) were effectively produced using Ni MOF in water with 42% glucose conversion. In contrast, only 9.73% of fructose and 8.2% sorbitol were produced with 31% conversion when using Zn MOF. This difference can be attributed to the larger surface area and active sites of Ni MOF, which allowed for greater interaction among the catalyst, glucose molecules, and hydrogen donor solvent, resulting in higher reaction rates [11]. The established understanding regarding the effective application of Raney nickel, alongside its prevalent selection for industrial-scale sorbitol synthesis utilizing this catalyst [3,4,14], as well as the analogous occurrence in sorbitol production facilitated by metal catalysts such as palladium (Pd), iron-nickel (Fe-Ni) [7], and ruthenium (Ru) [5,49,50,51], underscores the notion that despite both catalyst types possessing acidic active sites, nickel-based MOF demonstrated remarkable catalytic efficacy, and this is attributed to its capacity to selectively reduce the carbonyl group (C=O) of glucose to the hydroxyl group (C−OH), thereby yielding sorbitol.

**Scheme 1:**
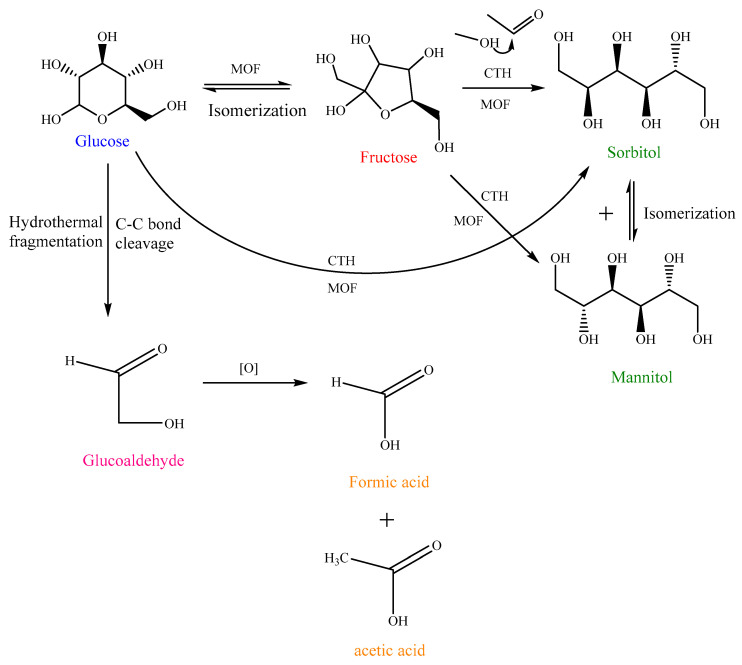
Proposed chemical transformations and degradation pathway that occurred in the presence of Ni/Zn MOF catalysts in a glucose–alcohol solution.

Nevertheless, the reduction of glucose was not the only transformation that occurred in the reaction medium; along with sorbitol, mannitol and fructose were also produced through side reaction pathways (Figure S7). Fructose was most likely generated by the isomerization of glucose, a process that can be facilitated by the active sites in the catalysts or simply by the thermal effect [49,52,53]. Mannitol, on the other hand, was not formed by the isomerization of sorbitol but through the hydrogenation of fructose [54]. Mannitol and sorbitol are alditols (sugar alcohols), meaning they contain only C−OH groups and no ketone or aldehyde groups, meaning the can be no direct isomerization of an alcohol to an alcohol without passing through a carbonyl intermediate, which is highly improbable under typical catalytic conditions [55]. All the potential chemical transformation reactions that can produce the obtained chemicals are depicted below as Scheme 1.

#### 2.8.2. Effect of Hydrogen Donors

The influence of the type of short-chain alcohol and diols used as a hydrogen donor was also explored and, as per Figure 4, it is evident that the existence of the two hydroxyl groups in the same molecule (diols) allows for a closer interaction between the hydrogen donor and the catalyst surface [56,57] compared to short-chain alcohols, and thus a higher extension of diols dehydrogenation provide more hydrogen to the reaction media. The observed rapid production of fructose from the use of isopropanol suggests a lack of performance from the carbon atom with a terminal OH group in hydrogen transfer reaction, and less conversion of glucose from the use of MOF catalysts suggests a lack of a strong Lewis acidity from the Zn MOF than Ni MOF; as a result, limited sorbitol yields (0.13% and 11.43%) were produced.

The use of diols provided fast conversion of glucose (~95.3%), together with a high selectivity toward sorbitol (~70–80%), with 1,4-cyclohexanediol producing 42.3% (Zn MOF) and 51.8% (Ni MOF), while 1,4-butanediol produced 38.42% (Zn MOF) and 45.12% (Ni MOF) of sorbitol with 89% glucose conversion, and 63–75% selectivity. This observation is attributed to the capability of 1,4-cyclohexanediol to undergo dehydrogenation to 1,4-cyclohexanedione, which is a very favorable reaction, later leading to a high hydrogen-donating capacity due to the presence of two hydroxyl groups, which can drive the glucose conversion to sorbitol [4]. The efficacy of these hydrogen donors follows a general trend where 1,4-cyclohexanediol > 1,4-butanediol > ethanol > isopropanol > water > catalyst in terms of hydrogen-donating ability and reaction efficiency.

## 3. Materials and Methods

AI Declaration: The authors declare that AI tools, such as Grammarly Pro version for the grammar of the write-up, and Topspin for NMR data processing. After using these tools/services, the authors reviewed and edited the content as needed and took full responsibility for its publication.

### 3.1. Materials

D-Glucose (≥99.5%), fructose (≥99%), sorbitol (99%), isopropanol (AR), 1,4-cyclohexanediol (99%), 1,4-butanediol (reagent plus^®^, 99%), ethanol (≥99.9%, GC and HPLC grade, LiChrosolv^®^), methanol (≥99.9%, GC and HPLC grade, LiChrosolv^®^), water (≥99.9%, GC and HPLC grade, LiChrosolv^®^), 2-mercaptobenzothiazole (2-MBT, for synthesis), potassium tert-butoxide (reagent grade, ≥98%), and mannitol (≥98%) were purchased from Sigma-Aldrich (Johannesburg, South Africa), while zinc(II)sulphate hexahydrate (ZnSO_4_·H_2_O, AR) and nickel(II)sulphate hexahydrate (NiSO_4_·H_2_O, AR) were purchases from Yaksha laboratories (Durban, KwaZulu-Natal, South Africa) and used without prior purification.

### 3.2. Synthesis of Catalysts

**Figure 5:**
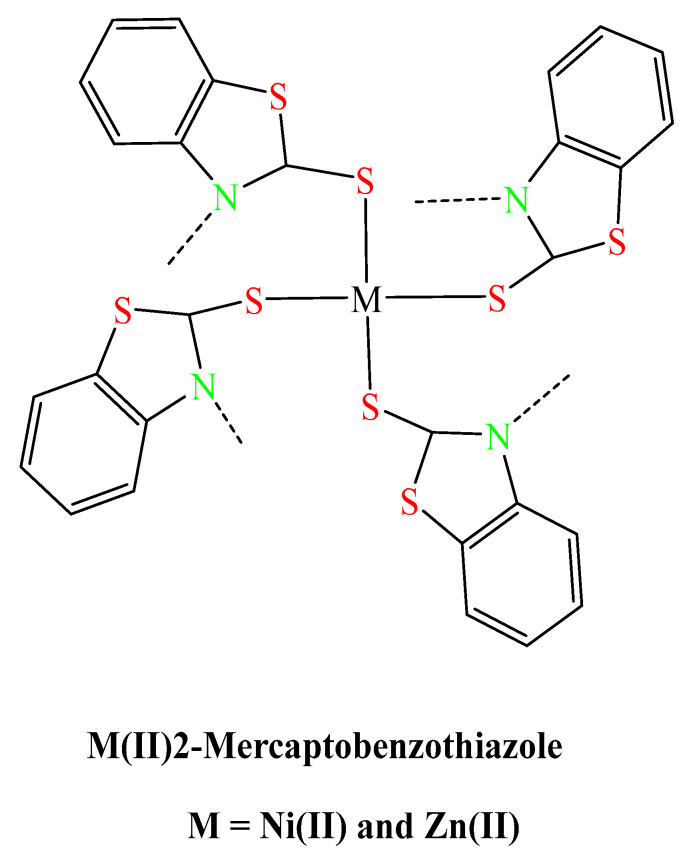
Proposed 2-MBT (M^1^ = Zn and M^2^ = Ni) based MOF unit structure.

An adjusted approach previously reported by Mphuthi, Erasmus [58] was utilized to create Ni and Zn MOFs (NiSO_4_·6H_2_O and ZnSO_4_·6H_2_O), where the required amount of metal salt (0.03 mol) was dissolved in water (30 mL) and the reaction solution was named solution A, followed by an organic linker (0.12 mol) being dissolved in 100 mL of MeOH and the mixture being named solution B. Solution B was transferred into a 250 mL RBF, followed by a dropwise addition of solution A while stirring constantly at room temperature. The reaction mixture was kept at room temperature for 24 h, after which the off-yellow milky (for Zn^2+^) and brown (for Ni^2+^) precipitates were obtained and separated by centrifugation (at 8000 rpm for 10 min), washed with ultra-pure water and methanol (20 mL for two runs), vacuum-dried, and calcined at 95 °C for 12 h. The obtained products (Figure 5) were further characterized.

### 3.3. Characterization Instrumentation

The conjugation systems (π, and n) and electronic transitions that normally show variable energy gaps of π→π*, n→π* transitions, metal to ligand or ligand to metal charge transfer transitions (ML or LMCTT) were studied using the electronic spectra acquired from UV–Vis absorption spectra (Genesy 50, UV-Vis, Thermofischer, Johannesburg, South Africa, Figure S1). The surface morphologies and chemical composition of Ni/Zn MOFs were observed using field emission scanning electron microscopy (FE-SEM, Zeiss Ultra Plus FEG SEM, Oberkochen, Germany), and elemental mapping was conducted using the energy-dispersive X-ray (EDX, Oxford X-max EDX detector, UK, Figure 1). The functional groups within the materials were analysed using Fourier Transform Infared spectroscopy (FTIR, Agilent Cary 360, Johannesburg, South Africa) in the scanning range of 650−4000 cm^−1^ at a resolution of 4 cm^−1^ and 120 scans per sample (Figure S2). The particle size distribution in liquid suspension was measured using a dynamic light scattering (DLS) using the Zetasizer (Nano ZS) instrument (Malvern Instruments Ltd., Malvern, UK), where approximately 0.5 g of the sample was dissolved in 10 mL of ultrapure water, sonicated for 10 min, and placed in the instrument for measurement. The recorded Z-average hydrodynamic diameter and particle distributions were calculated using the DLS instrument software version 7.13 (Figure S3). Textural properties were determined by N_2_ adsorption/desorption manometric porosimetry at 77 K using a Micromeritics Tristar II 3020 unit. Specific surface areas were calculated using the B.E.T. method, and the total pore volume was assumed to be that recorded at p/p0 for each sample (Figure S4). Acidity determination of Ni/Zn MOFs was performed using the pyridine poisoning method, and, after the experiment, samples were characterized by a Thermo Scientific Nicolet 6700 FTIR spectrometer (Figure S5).

Thermogravimetric (TGA) analysis was performed using the Mettler Toledo TGA/DSC1, ISF Model 1346 with STARe software version 9.20. In this, 10 mg of each sample was weighed and placed on the instrument with a temperature range of from 30 to 800 °C. The synthesized compounds were found to be thermally stable and can be utilized at elevated temperatures. Furthermore, the synthesized ionic compounds showed high electrostatic forces resulting in a relatively higher thermal stability, and the recorded thermograms are shown in Figure 2. The purity and crystallinity of the samples were characterized by powder X-ray crystallography (PXRD) using a Bruker D8 Advance model diffractometer. Measurements were taken in the 2*θ* range of 9–80°, using Cu-Kα1 radiation at a scan rate of 2°/min. The average crystallite size was determined based on Scherrer’s formula from the line broadening of the peak (Figure S6).

X-ray photoelectron spectroscopy (XPS, Figure 3) analysis was used to analyze the surface chemistry of the Ni MOF and determine elemental composition, chemical state, and surface contaminations. This was successfully achieved by utilizing the PHI Versaprobe that is powered by a proprietary high-flux X-ray source, producing a focused monochromatic X-ray beam that was used to irradiate the sample’s surface. The instrument was used to measure the kinetic energy of the emitted photoelectrons against the incoming X-rays to determine the binding energy of the electrons. This binding energy is unique to each element and its chemical state, and in this analysis a 100 μm diameter monochromatic Al Kα x-ray beam (hν = 1486.6 eV) was generated by a 25 W and 15 kV electron beam, and, from the recorded binding energies, a Multipack version 9 software was utilized to analyse the spectra to identify the chemical compounds and their electronic states using Gaussian–Lorentz fits. A low-energy Ar^+^ ion gun and a low-energy neutralizer electron gun were used to minimize charging on the surface. The binding energy calibration was achieved by using the high-energy peak of Cu 2p3 at 932.62 eV and the low-energy peak of Au 4f7 at 83.96 eV. The retard linearity was set to keep the difference between these two peaks constant at 848.66 eV, and the work function of the analyzer was set to 3.7 eV for the Ag3d5 peak to be at 368.27 ± 0.1 eV.

### 3.4. Determination of Acid Sites Using the Poisoning Test (Brønsted-Lewis)

Acid sites of the catalysts were determined using DRIFT spectroscopy combined with in situ adsorption of pyridine and 2,6-lutidine to evaluate their contributions towards total catalytic activity. The catalyst samples were finely ground in a mortar and activated at 110 °C overnight. The samples were transferred to different test tubes (in duplicates); in one set, 2 mL of pyridine was added and vortexed (Lewis), and, in another set, 2 mL of 2,6-lutidine was added and vortexed (Brønsted). Adsorption/equilibration with pyridine was then conducted at 105 °C, and at 120 °C for 2,6-lutidine. The bands at 2370–1435 cm^−1^ (coordinated pyridine) and 1040–740 cm^−1^ (coordinated 2,6-lutidine) were used to identify and quantify the Brønsted and Lewis acid sites, respectively [27].

### 3.5. Catalytic Tests

The CTH catalytic tests were performed in a 50 mL stainless-steel Parr benchtop reactor fitted with a temperature controller, a mechanical stirrer, and a pressure transducer to monitor the reaction conditions. In a typical assay, 2 g of D-glucose was dissolved in 25 mL of ethanol, isopropanol, 1,4-cyclohexanediol, or 1,4-butanediol, and the solution was loaded in the reactor vessel together with an appropriate amount of MOF catalyst. The reactor vessel was closed and continuously flushed with nitrogen to ensure inert conditions, pressure was kept at 6 KPa, and reactions were conducted at a temperature of 150 °C, 6 h of reaction time, and stirring at 100 rpm [4]. Autogenous pressure conditions (7 bar) were used in all experiments, and after completion, the reaction mixture was cooled down with an ice water bath, filtered, and the sample filtrate was characterized to monitor the conversion of the substrate to sorbitol.

### 3.6. Product Analysis

The analysis of the reaction filtrates was performed using high-performance liquid chromatography (HPLC). After each reaction, the sample pH was adjusted to a range of 2–3, filtered through a 0.22 μm syringe filter, and transferred to 1.5 mL high-performance liquid chromatography (HPLC) glass vials. The reaction products were detected and analyzed using HPLC (Shimadzu, Johannesburg, South Africa) equipped with a BioRad Aminex^®^- HPX-87H column (Lasec, Durban, South Africa), and 5 mM sulphuric acid. The operating conditions of the analysis were: column temperature–45 °C, flow rate—0.6 mL/min, injection volume—20 μL, and duration: 0–20 min. The products were quantified based on generated calibration curves, and the peak areas obtained were used to calculate conversion, selectivity, and the produced yields using the following Equations (1)–(3):

(1) $$Conversion(\%)=\frac{moles\;of\;initial\;glucose-moles\;of\;final\;glucose\;desired\;product}{moles\;of\;initial\;glucose}\;\times 100$$

(2) $$Selectivity(\%)=\frac{moles\;of\;desired\;product}{moles\;of\;initial\;glucose-moles\;of\;final\;glucose}\times 100$$

(3) Yield(%)=Conversion(%)×Selectivity(%)/100

## 4. Conclusions

The synthesis of Ni and Zn MOF catalysts was successfully achieved, and their characteristics were evaluated using several analytical methods, including UV-Vis, FTIR, TGA, BET, PXRD, DLS, SEM, and EDX. The synthesis of sorbitol by catalytic transfer hydrogenation employing short-chain alcohols and diols as hydrogen donors in conjunction with these catalysts was evaluated. This study demonstrated that both types of hydrogen donors had the potential to transfer hydrogen, with diols emerging as the superior hydrogen donors under the conditions tested. Furthermore, it has been established that glucose can participate in a disproportionation process, proving that sorbitol may be produced by self-reduction and catalytic transfer hydrogenation with sacrificial alcohol. Furthermore, when compared to previously used catalysts such as Raney-Ni for sorbitol production, the use of MOFs, particularly Ni-based MOFs, is a significant element that has the potential to improve sorbitol yield; however, important goals such as providing original catalysts with eco-friendliness, non-toxicity, high stability, and selectivity toward a target product remain unmet.

Another issue is to produce sugar alcohols more economically under low to medium working conditions while using less catalyst. The capacity to separate hexitols from one another or from leftover sugars or other generated by-products is necessary for the safe use of hexitols in the food, pharmaceutical, and cosmetic sectors, even though the materials utilized in this process have demonstrated potential. A large-scale sugar catalytic hydrogenation unit with several interconnected systems operating in concert should then be built based on bench-scale tests to assess the viability of commercializing this hexitol synthesis in an industrial context.

## Figures and Tables

**Table 1:** Hydrogenation of glucose to sorbitol over catalysts with non-noble metals, hybrid, and MOF-derived.

Catalyst Type	Specific Catalyst	Hydrogen Donor/Solvent	Temp. (°C)/Time (h)	Key Performance (Sorbitol Yield)	Reference
Non-Noble	Mo-Promoted Raney Ni	Ethanol, 1,4-Butanediol or 1,5-Pentanediol (Diols)	130/6	Sorbitol yield: 87.3% (with 1,4-butanediol); high selectivity and stability with diols over 550 h. The most common CTH system in alcohol media.	[4]
Non-Noble	Raney Ni	Methanol, ethanol, i-Propanol	130/6	∼58.2% sorbitol yield; feasibility demonstrated, but lower performance than with diols.	[3]
Non-Noble	Cu-Ni-Al_2_O_3_	1,4-Butanediol (Diol)	150/Continuous Flow	∼67% sorbitol yield; highlighted the efficiency of diol donors.	[33]
Bimetallic/Hybrid	Ni-Ru/Porous Carbon Sphere (Ni-Ru@PCS)	Water (Implied H_2_ or CTH with high performance)	140/2.5	100% selectivity at 99% conversion. (Though synthesis is complex, the result was outstanding).	[47]
Noble Metal	Ru Complexes (Azo-azomethine-based Ru(II))	i-Propanol	Not specified (Low-Pressure CTH)	Highly effective aldose reductase mimetics.	[48]
MOF-Derived	Ni(II)- or Zn(II)-MOFs	Water, ethanol, isopropanol, 1,4-butanediol, and 1,4-cyclohexanediol	150/6	∼51.8% sorbitol yield (with 1,4-cyclohexanediol); feasibility demonstrated, but lower performance compared to Raney Ni with diols.	This study

## Data Availability

All data are presented in the manuscript and available upon request.

## Supplementary Materials

The following supporting information can be downloaded at: https://www.mdpi.com/article/10.3390/molecules30234565/s1, Figure S1: UV-Vis spectra of (a) NiSO_4_.6H_2_O, (b) Zn(aca)2, (c) Ni MOF and (d) Zn MOF; Figure S2: Superimposed FTIR spectra of Ni and Zn MOFs; Figure S3: Particle size distribution of (a) Ni:2-MBT and (b) Zn:2-MBT MOFs; Figure S4: BET analysis: (a) Plot of N_2_ adsorption-desorption isotherm, (b) BET fitted data, (c) Ni MOF and (d) Zn MOF pore volume vs. pore width; Figure S5: Superimposed DRIFT spectra of adsorbed (a) pyridine and (b) 2,6-lutidine on Ni and Zn MOFs; Figure S6: XRD patterns of (a) Zn MOF and (b) Ni MOF; Figure S7: HPLC chromatograms obtained from the use of: (a) catalysts only, and (b) reaction mixtures.
