# Supplementary material for: Novel Ni/Zn MOFs for Sorbitol Production via Catalytic Transfer Hydrogenation
Source: Molecules. 2025 Nov 27;30(23):4565. doi: 10.3390/molecules30234565 (PMC12692985; doi:10.3390/molecules30234565)
Supplement: Supplementary file 1 [file molecules-30-04565-s001.zip › molecules-3897378-supplementary.pdf]

Novel Ni/Zn MOFs for Sorbitol Production via Catalytic Transfer Hydrogenation

Vuyolwethu Tokoyi * and Nirmala Deenadayalu

Department of Chemistry, Faculty of Applied Sciences, Durban University of Technology, P O. BOX 1334 Durban, South Africa; nirmalad@dut.ac.za
* Correspondence: vuyolwethut@dut.ac.za

Supplementary information

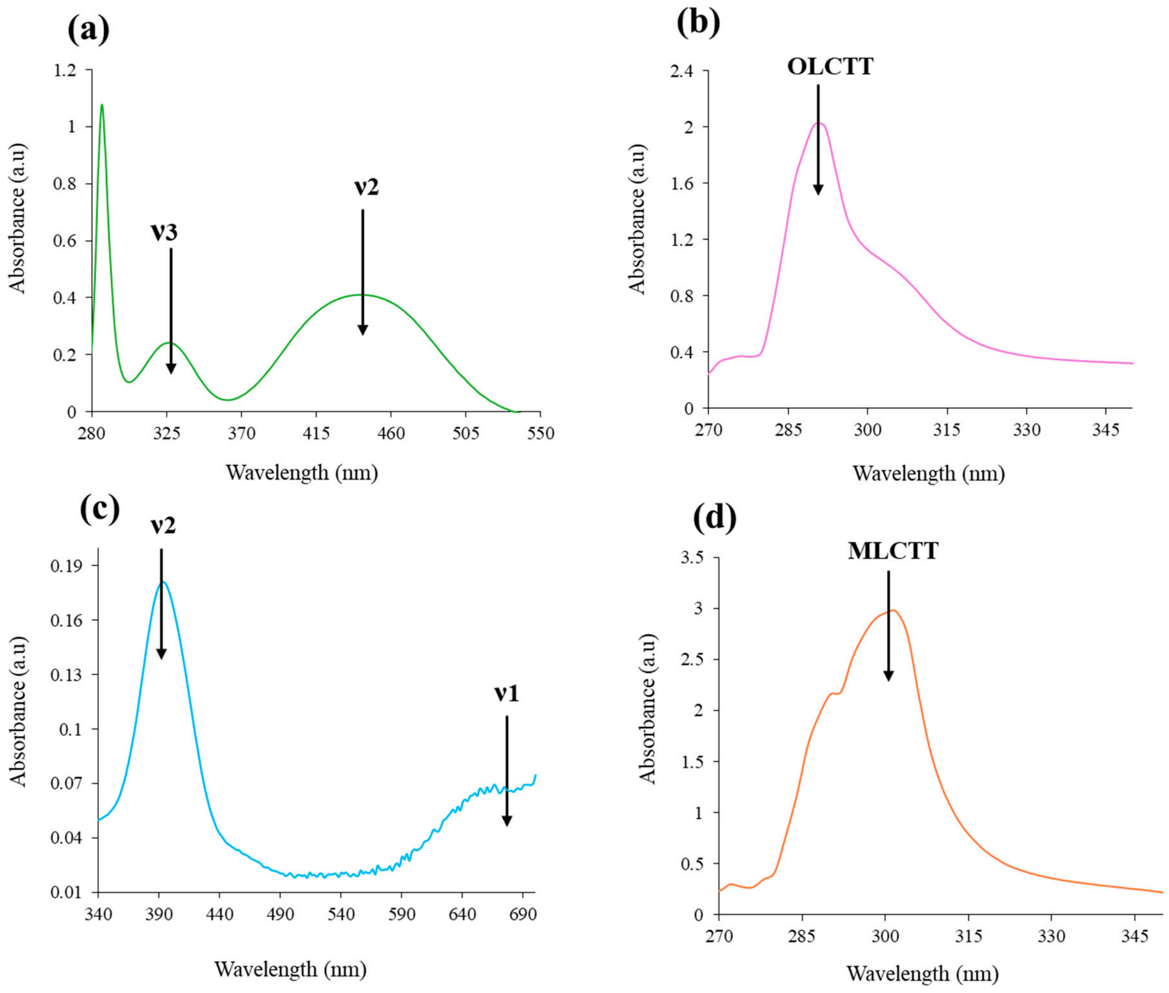

Figure S1: UV-Vis spectra of (a) $NiSO_4.6H_2O$, (b) $Zn(aca)_2$, (c) Ni MOF and (d) Zn MOF

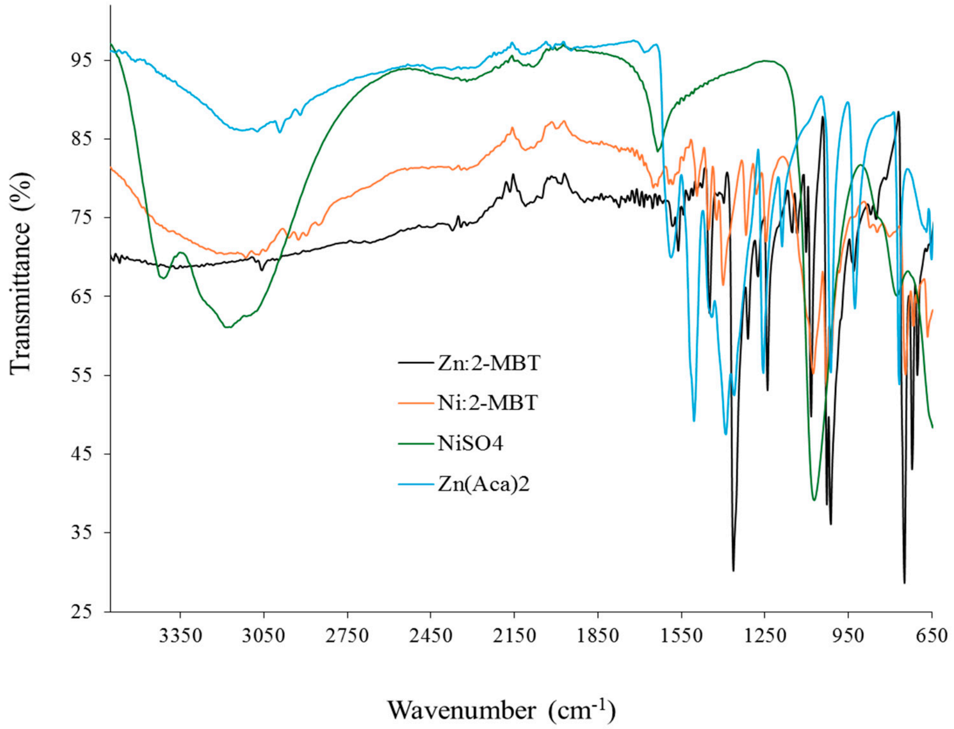

Figure S2: Superimposed FTIR spectra of Ni and Zn MOFs

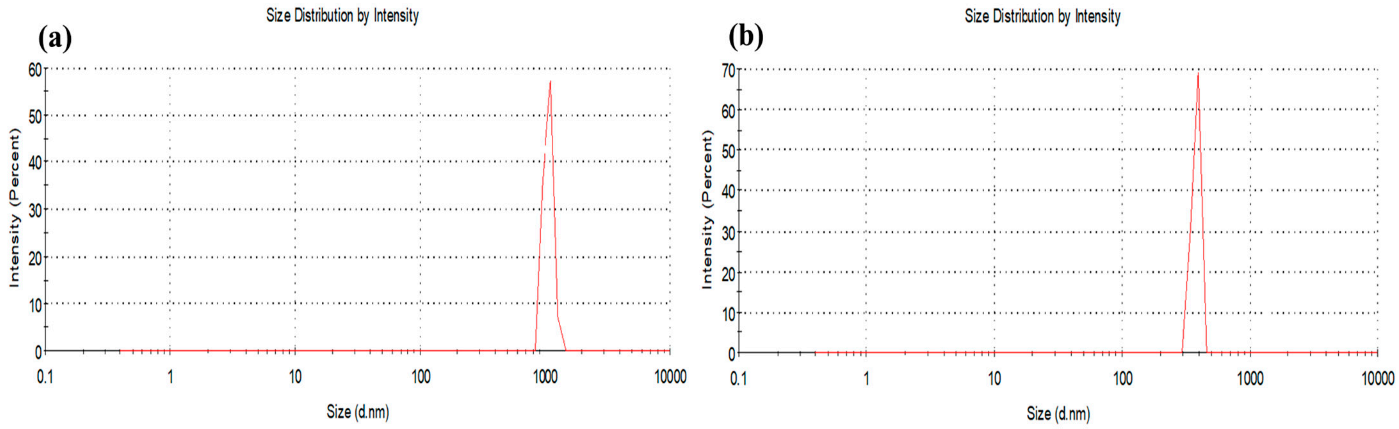

Figure S3: Particle size distribution of (a) Ni:2-MBT and (b) Zn:2-MBT MOFs

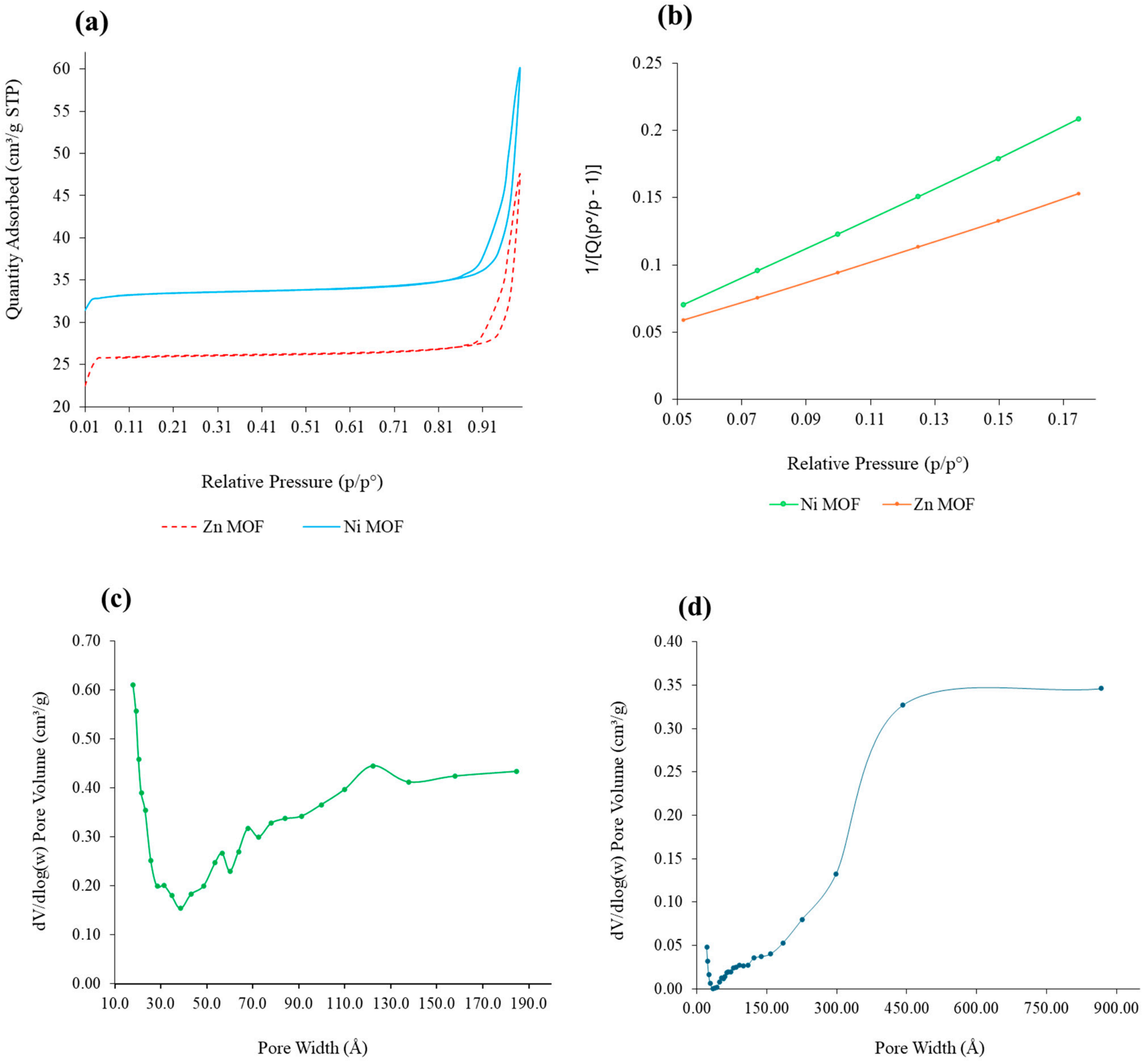

Figure S4: BET analysis: (a) Plot of N_2 adsorption-desorption isotherm, (b) BET fitted data, (c) Ni MOF and (d) Zn MOF pore volume vs pore width.

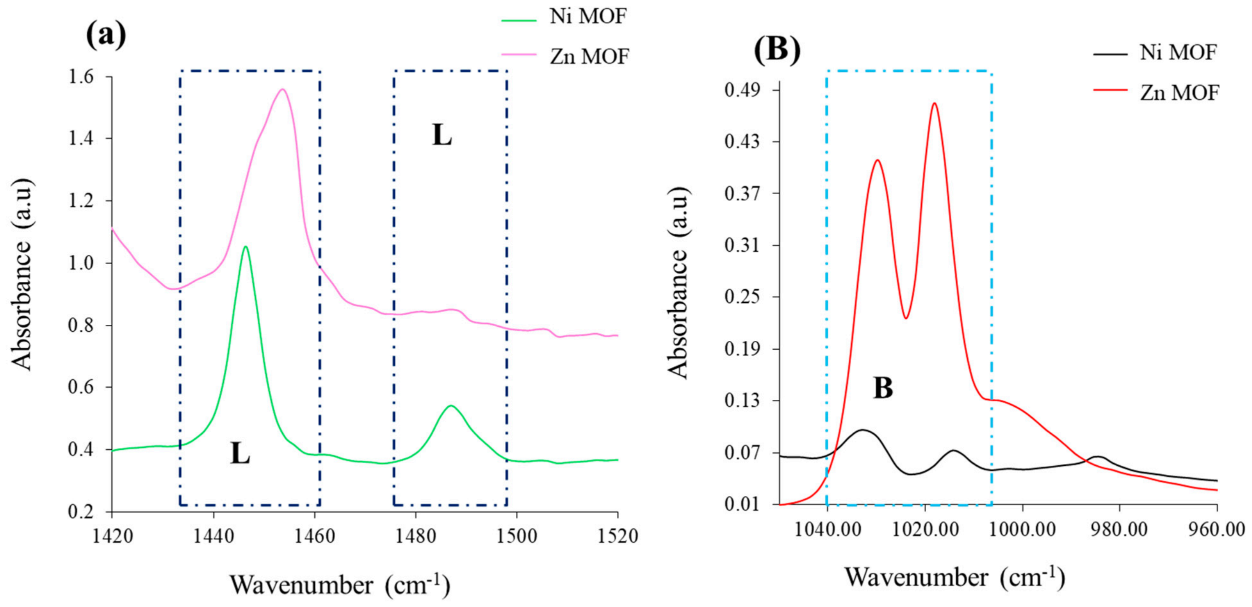

Figure S5: Superimposed DRIFT spectra of adsorbed (a) pyridine and (b) 2,6-lutidine on Ni and Zn MOFs

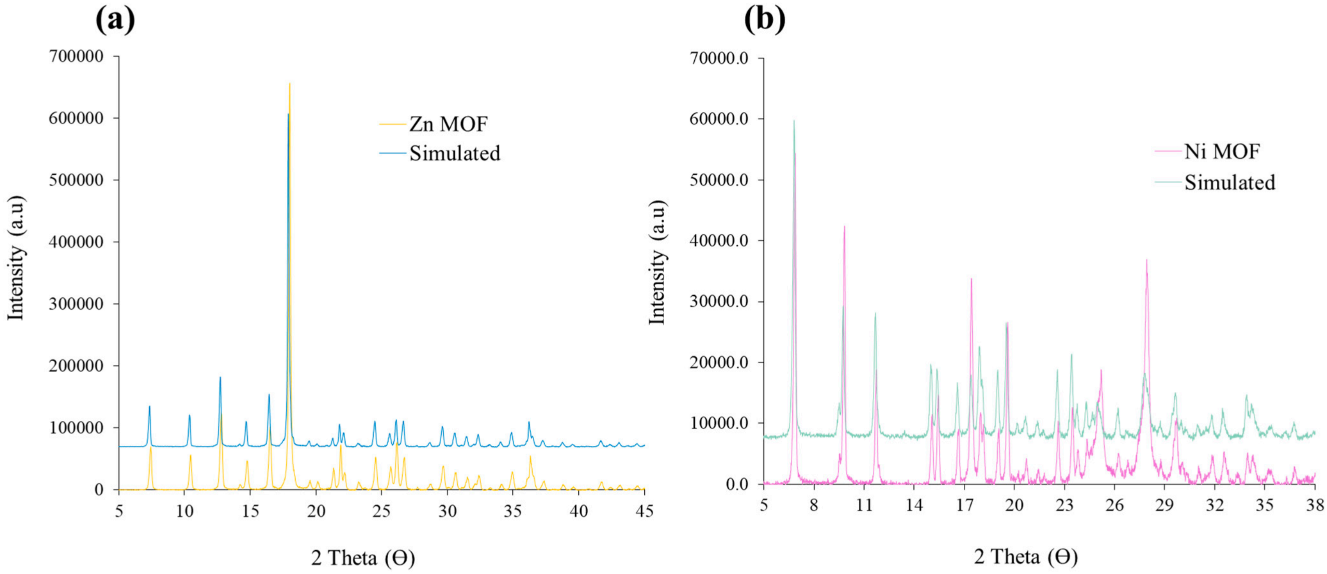

Figure S6: XRD patterns of (a) Zn MOF and (b) Ni MOF

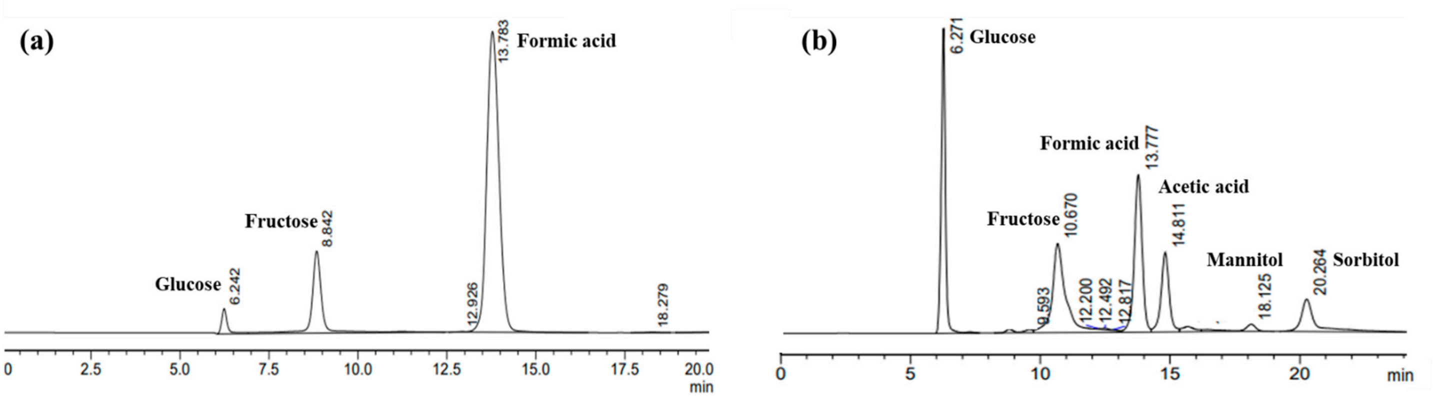

Figure S7: HPLC chromatograms obtained from the use of: (a) catalysts only, and (b) reaction mixtures
